# Foraging strategies are maintained despite workforce reduction: A multidisciplinary survey on the pollen collected by a social pollinator

**DOI:** 10.1371/journal.pone.0224037

**Published:** 2019-11-06

**Authors:** Paolo Biella, Nicola Tommasi, Asma Akter, Lorenzo Guzzetti, Jan Klecka, Anna Sandionigi, Massimo Labra, Andrea Galimberti

**Affiliations:** 1 University of Milano-Bicocca, Department of Biotechnology and Biosciences, Milan, Italy; 2 University of South Bohemia, Faculty of Science, Department of Zoology, České Budějovice, Czech Republic; 3 Czech Academy of Sciences, Biology Centre, Institute of Entomology, České Budějovice, Czech Republic; University of California San Diego, UNITED STATES

## Abstract

The way pollinators gather resources may play a key role for buffering their population declines. Social pollinators like bumblebees could adjust their foraging after significant workforce reductions to keep provisions to the colony optimal, especially in terms of pollen diversity and quantity. To test what effects a workforce reduction causes on the foraging for pollen, commercially-acquired colonies of the bumblebee *Bombus terrestris* were allowed to forage in the field and they were experimentally manipulated by removing half the number of workers. For each bumblebee, the pollen pellets were taxonomically identified with DNA metabarcoding of the ITS2 region followed by a statistical filtering based on ROC curves to filter out underrepresented OTUs. Video cameras and network analyses were employed to investigate changes in foraging strategies and behaviour. After filtering out the false-positives, HTS metabarcoding yielded a high plant diversity in the pollen pellets; for plant identity and pollen quantity traits no differences emerged between samples from treated and from control colonies, suggesting that plant choice was influenced mainly by external factors such as the plant phenology. The colonies responded to the removal of 50% of their workers by increasing the foraging activity of the remaining workers, while only negligible changes were found in diet breadth and indices describing the structure of the pollen transport network. Therefore, a consistency in the bumblebees’ feeding strategies emerges in the short term despite the lowered workforce.

## Introduction

Social pollinators, such as bumblebees, are subjected to multiple stressors that ultimately cause population reductions. These declining trends are mostly due to climate change [1,2] and several “pollinator-unfriendly” practices related to agriculture (i.e., the use of monocultures, the use of harmful agrochemicals [3,4], and the use of synthetic fertilisers causing both shifts in the vegetation and disappearing of flowers the bees forage on [5]). Moreover, land use change [6], the lack of flower diversity [7] (e.g. overgrazing or frequent mowing [8]), the reduction of natural ecosystems nearby fields [9], the spread of parasites and diseases [10], and the overwhelming competition from domesticated bees [11,12] also impact the dynamics of bumblebees and other pollinators’ populations. Several of these factors may lead to a significant reduction of workforce in a colony with important implications in the short term (feeding the developing larvae [13]) and in the long term (colony fitness [14]).

Gathering sufficient and appropriate resources is a key nutritional aspect for stabilizing pollinator populations [15–18], and could be severely disrupted by reduction of the number of workers in colonies of social pollinators. For example, task allocation could be modified between workers in a colony after a drop in workforce size [19]. However, it is not known how drops in the extent of the foraging workforce could modify selectivity for resources and foraging effort of pollinators. Two main expectations could be drawn specifically regarding changes in the foraging, after a workforce reduction. Firstly, the remaining workers could increase their foraging activity to maintain the total amount of resources brought to the nest. This expectation could result either from the individual bumblebees making more foraging trips or from a colony allocating a higher number of workers to the task of foraging. These are supported by studies showing that the removing half of the bumblebees workforce can alter task allocation in the colony [20] and also that the rate of larval feeding by the workers is increased inside the nest [13]. Thus, a similar compensation could also be expected in relation to the foraging activity outside the nest. Secondly, a change in the composition of resources collected by individual workers could occur in response to a shift in competitive interactions. According to the Optimal Foraging Theory [21,22], selectivity for resources is influenced not only by their energetic value but also by competitive interactions, as a higher density of foragers causes faster depletion of the resources and triggers a wider diet breadth in the foragers as a consequence (i.e. density-dependent mechanisms [23,24]). On the other hand, a significant loss of workforce in a social pollinator colony could decrease the competition for resources in the vicinity of the nest and the remaining workers would then be predicted to become more selective and focus on the most rewarding plants. To our knowledge, this hypothesis has not yet been tested in the field, but laboratory experiments showed that individual bumblebees adjust their selectivity according to the level of intra-specific competition and to changes in colony size [23,25]. Both expected responses, i.e. increased foraging activity and changes in resource selectivity, could lead to maintaining the resource intake by the pollinator’s colony after the workforce reduction.

In the case of pollen collected by pollinators, studying insect-plant interactions is complicated by several methodological aspects. In addition to the direct observation of an insect’s behaviour [26], the analysis of pollen on an insect’s body can reveal the interactions that happened during a pollinator’s trip and can also yield the rarest interactions that normally remain undetected during observational surveys [27,28]. Yet, morphology-based identification of pollen lacks a uniform discriminatory power and requires great taxonomical knowledge [29–31]. However, the potential benefits of pollen studies highlight the need to improve methods that are alternative to the morphological analyses. In this context, DNA-based approaches, such as DNA barcoding and DNA metabarcoding, represent a reliable alternative [32,33]. In other words, by using integrative approaches (e.g. DNA metabarcoding applied to ecological questions), methodological issues with pollen identification can be overcome and the interactions and the resource usage by declining pollinators can be explored in more depth.

Here, we tested the expectations listed above about changes in foraging due to colony workforce reduction by experimentally inducing a sudden decline in the colony size of commercial colonies of the bumblebee *Bombus terrestris* (Linnaeus, 1758). We focused on bumblebees, because they are among the most effective pollinators [34], native to several regions of the world (while honeybees are often domesticated [35]) with several species in population decline[1,10], and, they depend on the habitat they live in because the colonies need a high amount of resources for fitness [2]. In this study, we intended to recreate a situation of workforce loss due to natural or human-based environmental conditions, by removing half of the workforce. Such a removal is supported by studies showing that a colony’s workforce can face reductions up to 50% due to several stressors, such as pathogens and parasites[36,37], or pesticides[38], or the combination of pesticide exposure and poor nutrition[39]. We explored the foraging behaviour before and after the manipulation with video recordings and also compared resource utilization based on pollen identification with a DNA metabarcoding approach. Our specific aims were to investigate the effect of an experimental reduction of the bumblebees’ workforce by focusing on short-term responses (i) in the foraging strategies of individuals and in the associated bumblebee-plant networks, (ii) in the foraging rate per unit of time, and (iii) in the diversity of the collected plants and in plant’s traits of pollen production. This experiment has the potential of providing new insights into the ways social pollinators respond to environmental disturbances (e.g. causing decline as those indicated above) by interacting with plant resources within the context of pollination ecosystem services.

## Material and methods

### Study area, experimental set-up, and sample collections

The experiment was conducted in a meadow near Český Krumlov, 18 km southwest of České Budějovice (Czech Republic, 48°49'30.52" N, 14°19'4.02" E), that belongs to a 62 ha natural area located at an altitude of 600 m a.s.l. and consists of forest, isolated trees, and shrubs, while a portion is covered by species rich calcareous grasslands managed by occasional extensive grazing. Around this zone, a mosaic of agricultural areas and urban settlements occurs. The study site is part of a publicly accessible area where sample collections are allowed (with the exception of species protected by law). The experiment and the collection of samples were carried out on sunny days without strong wind or rain, in summer 2016.

Four commercial colonies of the bumblebee *Bombus terrestris* were bought from a private company (Koppert s.r.o., Nove Zamky) and were placed in pairs at the study site at a distance from each other of about 500 m in order to capture possible minor changes in floristic composition. All colonies were marked and placed in the field under shade to prevent overheating. The number of used colonies lies within the range used in other studies about bumblebees foraging [40–43]. In each pair, a colony was used as a control, and it was not treated during the length of the experiment, while a second colony was used to apply a treatment of diminishing the worker population, which in practice consisted of manually removing 50% of the workers relative to the number of workers present in the period before removal in that colony. This removal threshold was inspired by studies reporting mortalities or worker losses up to 50% with respect to control colonies due to multiple stressors (see [36,38,39]). For removing the workers, as we used nest boxes with a way-in and a way-out holes, the way-in was left open for an entire afternoon so that workers could return to the nest but none could leave it, and then the nest was completely closed during the following night. Early in the next morning, light anaesthetization with CO_2_ was applied to the nest for a very short time, workers were counted and half of the worker amount was removed from the nest.

Four days after placing the colonies in the field, the workers’ pollen pellets were collected from the corbiculae of the legs just before entering the nest and after light anaesthetization with CO2 [44] (the workers were afterwards released outside their nest to avoid immediate complications for the larvae related to workers being anesthetized [45]). The pollen of 18 bumblebee workers for each nest were surveyed before workforce halving (“before” phase, 6th-11th July). In the period after removing the workers (“after” phase, 20th-23rd July), pollen pellets of 18 workers for each colony were collected in the same way as the “before” period (17 workers for one of the nests). There was a low chance of resampling individual bees, given the large size of the colonies that were used: there were an average of 160 workers before removal. The number of samples collected was similar to other studies on DNA metabarcoding of pollen [46,47]. Pellets were collected with sterile tweezers and placed in Eppendorf tubes, marked with codes and stored in a freezer at -20°C. The number of samples included in the analyses provided a plant diversity per nest that was estimated to be 83% and 78% of the asymptotic plant diversity of each treated nest as shown by the Chao2 estimator calculated by the iNEXT package of R with incidence data (±4 species in nest 1 and ±4.8 species in nest 2).

Local botanists provided an accurate check-list of the flowering plant species at the study area (i.e., 112 plant species, see S1 Table). Those species that were not available in public nucleotide databases (i.e., NCBI and BOLD) were sampled (i.e., 54 plant species, one or two young leafs each, stored at −20°C) to create a complete DNA barcoding reference dataset. Reference ITS2 sequences for the remaining species were directly retrieved from GenBank NCBI prior to accurate validation of the accessions (i.e., availability of voucher details and complete overlapping with the DNA barcoding region sequenced in the bumblebees’ pollen pellets). Overall, the final reference dataset encompassed 1196 ITS2 sequences.

### DNA analyses and taxonomical assignments

Reference ITS2 DNA barcodes for the sampled plant species were obtained as described in [48] and deposited in EMBL GeneBank under the accessions reported in S1 Table.

The pollen samples were analysed as described in S1 Appendix. In brief, one pollen pellet for each bumblebee was grinded and the DNA was extracted according to standard protocols. A HTS (High-throughput sequencing) DNA metabarcoding approach was used to analyse these DNA extracts, by targeting the nuclear internal transcribed spacer 2 region (ITS2) [32,33,49] with S2F and S3R primer [50]; before amplification, DNA extracts concentration was normalized by means of quantitative real-time PCR as described in [51]; amplification was performed with PCR, and Index PCR and library sequencing were performed through the Illumina MiSeq. The raw reads were paired, pre-processed and filtered according to the standard bioinformatics pipeline and OTUs (Operational Taxonomic Unit) were obtained by clustering reads with a 99% sequence identity; these OTUs were assigned to a plant species or to a genus [52]. All details on sample preparation, sequencing, bioinformatic analysis and taxonomical assignment are described in S1 Appendix.

### Selection of OTUs

Sorting false positives from data produced with DNA metabarcoding has been recently underlined [53]. In order to exclude false-positive OTUs from the dataset, the ROC (Receiver Operating Characteristic) framework was used to quantify a trade-off of acceptance or rejection of OTUs within the analyzed pollen samples. The ROC framework assesses the true positive rate and the true negative rate of a test [54], based on the Youden index. This approach can improve the reliability of OTU assignments by establishing defensible thresholds for rejection or acceptance [55]. This is a well-accepted methodology for threshold detection, since it is used in several biological fields, including DNA- and environmental DNA-based studies [55,56].

In the samples of this study, some OTUs were represented with only a very low number of reads. This would hint at the presence of false positives. Therefore, in order to find reliable thresholds, we followed the suggestions of [55] which employs ROC curves instead of arbitrarily cut-off values for excluding OTUs from the samples.

Specifically for each sample independently, a categorical variable “negative” was assigned to the OTUs with 0 number of DNA reads and “positive” was assigned to the OTU with reads >0. A GLM (Generalized linear regression) with an overdispersed Poisson distribution (quasipoisson) was performed independently on each sample in order to estimate the distribution of reads related to positives and negatives; the amount of reads per OTU was the response variable and "positive" or "negative" was the predictor variable. On the values estimated by the regressions, the *pROC* package [57] in the R environment [58] was used to estimate the per-sample cutting threshold and thus to identify which OTUs were false positives. Those OTUs with a number of reads below the estimated thresholds were excluded from the dataset and considered as false positives. The resulting dataset was used in the following analyses (S2 Table and S1 Dataset).

### Networks of foraging

For each nest and at each experimental phase (time “before” and “after” removal of workers), *i*-*sp* matrices representing the interactions of individual bumblebees and plant species were analysed to investigate changes in the foraging strategies by means of networks analyses (data in S1 Dataset), an approach equivalent to what used in [59,60]. Network analysis is an efficient way of studying interactions, because, rather than focusing uniquely on diversity and richness, it employs a set of indices with a clear ecological interpretation aimed at describing the network of interactions among organisms: it highlights who interact with whom, the interaction overlaps with other species/individuals of the same trophic level, the competition and specializations patterns [61]. Network analyses are very practical because they can explore patterns based on interactions of multiple individuals (network level indices) or can be focused on the level of single individuals (individual level indices). In our study, both binary and quantitative matrices were used, because different aspects are accounted for; The binary ones are useful for studying network structures where all links are equal (as they are based on the presence and absence of interactions), while in the quantitative ones, the links have different weights according to the intensity of each interaction (e.g. interaction frequency, transferred biomass, etc).

Firstly, we tested several node-level indices (where a “node” is either a foraging bumblebee or a plant species). Specialization was investigated using: (a) the “degree”, that is the number of plant species found in a pollen pellet; (b) RR, the “resource range”, that estimates the fraction of used resources to the total available[62] and is computed here as $1-\frac{R-r}{R-1}$, where *R* is the available resources (= plants) and *r* is the used plants; (c) PG, the “proportional generality”, is the quantitative diversity of consumers in relation to the potential resources; it is computed as the ratio between the power of the quantitative Shannon diversity *H* for consumers *p* and that for the abundances of resources *q*: $e^{H_{p}}/e^{H_{q}}$; (d) PDI, the “Paired Difference Index”, is the quantitative counterpart of RR and it compares the strongest quantitative interaction with all remaining interactions [63]; it characterizes the decay of performance as drift from the optimal resource; it is calculated here as $1-\frac{\sum_{i=2}^{R}\left(P_{max}-P_{i}\right)}{R-1}$, where $P_{max}$ is the maximum of all quantitative interactions, *P*_*i*_ is the quantity of interaction with the *i* plant, and *R* is the number of available resources (= plants); (e) *d’* index, which is a measure of specialization based on niche overlap among nodes[64] and is calculated as $\sum_{j=1}^{R}\left(p_{ij}^{'}\;ln\frac{p_{ij}^{'}}{q_{j}}\right)$, where *R* is the number of resources, $p_{ij}^{'}$ is a species’ *i* interaction with partner *j* as proportion of the sum of interactions of *i*, *q*_*j*_ is the sum of interactions of partner *j* divided by the total of all interactions. In addition, we studied the importance of plant species in the foraging network with the (f) “closeness centrality”, which indicates how a plant is near the core of the interactions based on the path lengths of the network; $CC=\;\frac{R-1}{\sum_{i:i\neq \text{v}}d\left(v,i\right)}$, where *R* is the available plants and *d(v*,*i)* is the geodesic distance between plant *v* and *i* [65]. Indexes (a), (b), and (f) are calculated from the binary interaction matrices (presence or absence of a plant in a sample), while indexes (c), (d), and (e) are based on the quantitative interaction matrix including the number of DNA reads of a certain plant species in a pollen pellet. Using DNA reads as a proxy of a quantitative amount of pollen was decently supported in Bell et al. (2018) and was already applied to networks in Pornon et al. (2017); these indexes include normalizations by matrix total.

For testing changes in these node-level indexes, each one was analysed with generalized linear mixed-effect models with library *lme4* [66] in the R environment with a given index as response variable, and the experimental phase (“before”, “after” workers removal), whether the colonies were treated or control (T./C.) and the interaction between experimental phase and T./C. as predictor variables. The nest identity was the random intercept. Poisson distribution or Gamma distribution with the log link function were used, depending on the response variable.

Secondly, to test whether the entire bumblebee-plant networks changed after the treatment, the interaction matrices included either binary interaction matrices or the count data of the DNA reads, such as in [28], standardized by the total of the matrix. For each nest, the network structure was studied by focusing on several aspects of networks. Firstly, the proportion of realized interactions was studied with (a) Link density LD [67], which is a quantitative measure of the proportion of realized interactions weighted by interaction diversity and is computed as $LD=\frac{1}{2}\left(\sum_{j=1}^{s}\frac{b_{j.}}{b_{.\;.}}2^{H_{q}}+\sum_{i=1}^{s}\frac{b_{.j}}{b_{.\;.}}2^{H_{p}}\right)$, where *s* is the number of species in the networks, $b_{‥}$ is the total sum of the matrix, *b*_*j*_ is the sum of the interactions of bumblebees *j* and *b*_.*j*_ is the sum of the interactions of plant *i*, *H*_*q*_ is calculated as $–\sum_{j=1}^{s}\frac{b_{j}}{b_{j.}}log_{2}\frac{b_{j}}{b_{j.}}$ with *b*_*j*_ as an interaction (and similarly for plants *H*_*p*_ and plant species *i*); (b) Connectance C [67], which is the proportion of realized links in the network and is calculated as $C=\frac{L}{I*J}$, *L* is the number of interactions, *I*, and *J* is the number of plant and animal species, respectively, and can vary from 0 to a maximum of 1. In addition, how resources are distributed among nodes was investigated with the nestedness index, so that in a nested network the generalist pool interacts with both specialists and generalists. It was calculated as (c) Nestedness based on Overlap and Decreasing Fill (NODF) and (d) the weighted counterpart WNODF[68], is based on decreasing fill and on paired overlap on the matrix. Between pairs of columns and pairs of rows, it detects the degree of nestedness *N*_*p*_ by comparing the marginal totals and the proportion of filled matrix cells located at the same position. Thus, for a matrix with *i* plants and *j* bumblebees, ODF=∑Np[i(i−1)2]+[j(j−1)2]. It ranges from 0 to 100 (fully nested). Moreover, the tendency of the network to divide into compartments, with implications for resource accessibility and competition, was calculated as (e) Modularity *Q*, and (f) the quantitative counterpart *Q*_*w*_, computed by the algoritm *DIRTLPAwb+* [69]; *Q* is computed as $\frac{1}{m}\sum_{i=1}^{r}\sum_{j=1}^{c}\left(A_{ij}-\frac{k_{i}d_{j}}{m}\right)\delta \left({\text{g}}_{\text{i}},{\text{h}}_{\text{j}}\right)$, where *A*_*ij*_ is the interaction matrix of *r* rows and *c* columns, *m* is the number of links, *k* is the node degree for a plant with label *h*, and *d* is the node degree for a bumblebee with label *g*, while the Kronecker function $\delta \left({\text{g}}_{\text{i}},{\text{h}}_{\text{j}}\right)$ is 1 if nodes *i* and *j* belong to same module or 0 otherwise. *Q* and *Q*_*w*_ range from 0 to its maximum of 1. Network-level specialization was also investigated. A niche-overlap measure of specialization of network-level interactions was studied with (g) Interaction Diversity $H_{2}^{'}$. It is computed as $\sum_{i=1}^{r}\sum_{j=1}^{c}\left(p_{ij}*lnp_{ij}\right)$, with *r* and *c* referring to rows and columns of the interaction matrix between a plant species *i* and pollinator species *j*, and *p*_*ij*_ is the proportion of the number of interactions in relation to the respective row total. Its possible maximum and minimum are obtained from the distribution of interaction totals of the matrix and used to normalize the index to vary between 0 and 1 (perfect specialisation) [64]. Specialization was also studied with (f) Generality and (g) Vulnerability indexes, that are the mean effective numbers of partners, that is of plants for bumblebees (Generality G) and of bumblebees for plants (Vulnerability V), weighted by the marginal totals; they are calculated as $\frac{1}{l/s}\sum_{j=1}^{s}b_{ij}$, where a node *i* is interacting with a node *j*, and *b*_*ij*_ is the sum of quantitative interactions between *i* and *j*, and the total number of links in the network is *l* and that of nodes is *s* [67].

Changes in these indices of network structure were tested by means of random permutations of the data, which test whether the difference between the observed networks is significant with respect to random expectations. To reach this goal, the interactions (matrix cells) of both networks were swapped randomly between the two networks (“before”, “after”), following [70,71], for 10000 times for each of the two networks. After each swap, the value of the difference was recalculated. The statistical significance was obtained by comparing the observed difference to the distribution of differences from the random permutations.

The node and the network indices were calculated with the packages *bipartite* [72] and *vegan* [73] in R.

### Rate of workers leaving the nest

All colonies (treated and control) were recorded with video cameras (Canon Legria HFR56) for a sample of three hours during a day during the experimental phases before removing workers and after removing workers. The camera was placed near the entrance of the nests, so that the number of leaving and of returning workers could be counted.

For testing changes in the foraging rate, the number of workers leaving during each 20 minute interval time was used as response variable (a total of 39 time units were used) in generalized linear mixed-effect models with library *lme4* in the R environment. The experimental phase was the predictor variable (i.e., the period “before” and “after” worker removal), and the nest identity was the random intercept. Poisson distribution with the log link function was used. A statistical offset term with the number of workers leaving the control nests per 20 minute time was included in the analyses due to the assumptions of the Poisson distribution, but it is considered equivalent to dividing the number of leavings in the treated colonies in a given unit of time by the number of leavings in the control colonies in the same time unit; this was performed in order to account for variations of foraging rate independent from the treatment.

### Plant diversity in the pollen pellets

To investigate changes in plant species composition in the pollen samples during the experimental time, a PER-MANOVA (Permutational Multivariate Analysis of Variance Using Distance Matrices) was used with the function *adonis* in the *vegan* package in R. Samples-per-plants matrices with presence / absence of a given plant in a given sample was considered the response variable, while the experimental phase (“before” and “after” workers removal), nest identity (nests 1 to 4), whether the colonies were treated or control (T./C.) and the interaction between experimental phase and T./C. were predictor variables.

### Traits of pollen production

Values of the plant trait of pollen production (“pollen quantity”) were assigned to both used and unused plants flowering at the study area during the experimental time (S1 and S2 Tables). These values were extracted from [74], which ranks plants from low (“P0”) to high (“P5”) levels of pollen quantity in several European plant species. Specifically, this ranking is based on the amount of pollen produced by the plant species, it is coherent with other rank-based pollen databases (see [75]) and it also is incorporated in the plant trait databases provided by the *TR8* package for R [76]. FThe probability of collecting pollen of a species was analyzed using logistic regression (generalized linear mixed-effect models) with presence/absence of a plant in a sample for a given colony as a response variable; the experimental phase (“before” and “after” workers removal), the (numerical) pollen quantity, whether the colonies were treated or control (“T./C.”), the interaction between experimental phase and pollen quantity and T./C. were the predictors; the nest identity was the a random intercept. The binomial distribution and logit as link function were used. The *lme4* package for R was employed. Confidence intervals were estimated with 1000 bootstrapping using the function *bootMer*.

## Results

### Sequencing, filtering, and taxonomic assignment of pollen loads

Illumina sequencing of pollen samples yielded 18,473,760 raw reads. After pair-ending and quality filtering, 5,600,000 reads were included in the dataset, and they were clustered in 167 OTUs, 51 of which showed high similarity with fungi accessions and were excluded from the dataset. The remaining OTUs were assigned to 44 plant taxa and specifically 90 OTUs (53.9%) to the species level and 26 OTUs (15.5%) to the genus level. The ROC filtering excluded 25 additional OTUs (at least 10 plant species) and a total of 72,361 reads were removed from the dataset with a mean of 36 reads per sample (with across sample st. dev. of 101 reads and a range of 0–1214 reads) which corresponds to an average of cutting thresholds across samples of 2.28% of the reads. Therefore, the filtered list of plant species encompassed 34 taxa (91.2% with species identity) with a mean of 2.25 taxa per sample, st. dev. = 1.54, min. 1 and max. 10 (S2 Table and S1 Dataset), corresponding to 3,392,081 total reads.

Ten taxa in the post-ROC dataset were not initially included in the floral checklist. Monofloral pollen pellets were 37% (53 samples), while 63% (90 samples) were polyfloral (44 pollen samples of two plant taxa, 46 samples of more than two taxa).

### Pollen plant diversity, node- and network-level responses to the treatment

Taxa composition of the pollen samples changed over the study period (S1 Fig). In particular, the experimental phase (before/after workforce reduction) predicted the plant identity of the pollen samples better than the nest identity, although both variables were significant (Table 1); conversely, no significant differences were found between treated and control colonies.

**Table 1:** Results from a PER-MANOVA statistics testing the effect of the experimental phase (before and after workers removal), nest identity (nest 1 to 4) and T./C. (treated and control colonies) in the presence/absence of plant species in the pollen pellet samples. Significant results are highlighted in bold.

Variable	Df	R^2^	p
Experimental phase	1	0.104	**<0.001**
T./C.	1	0.007	0.325
Nest Id.	2	0.064	**<0.001**
Experimental phase *x* T./C.	1	0.005	0.583
Residuals	137	0.821	
Total	142	1	

**Fig 1:**
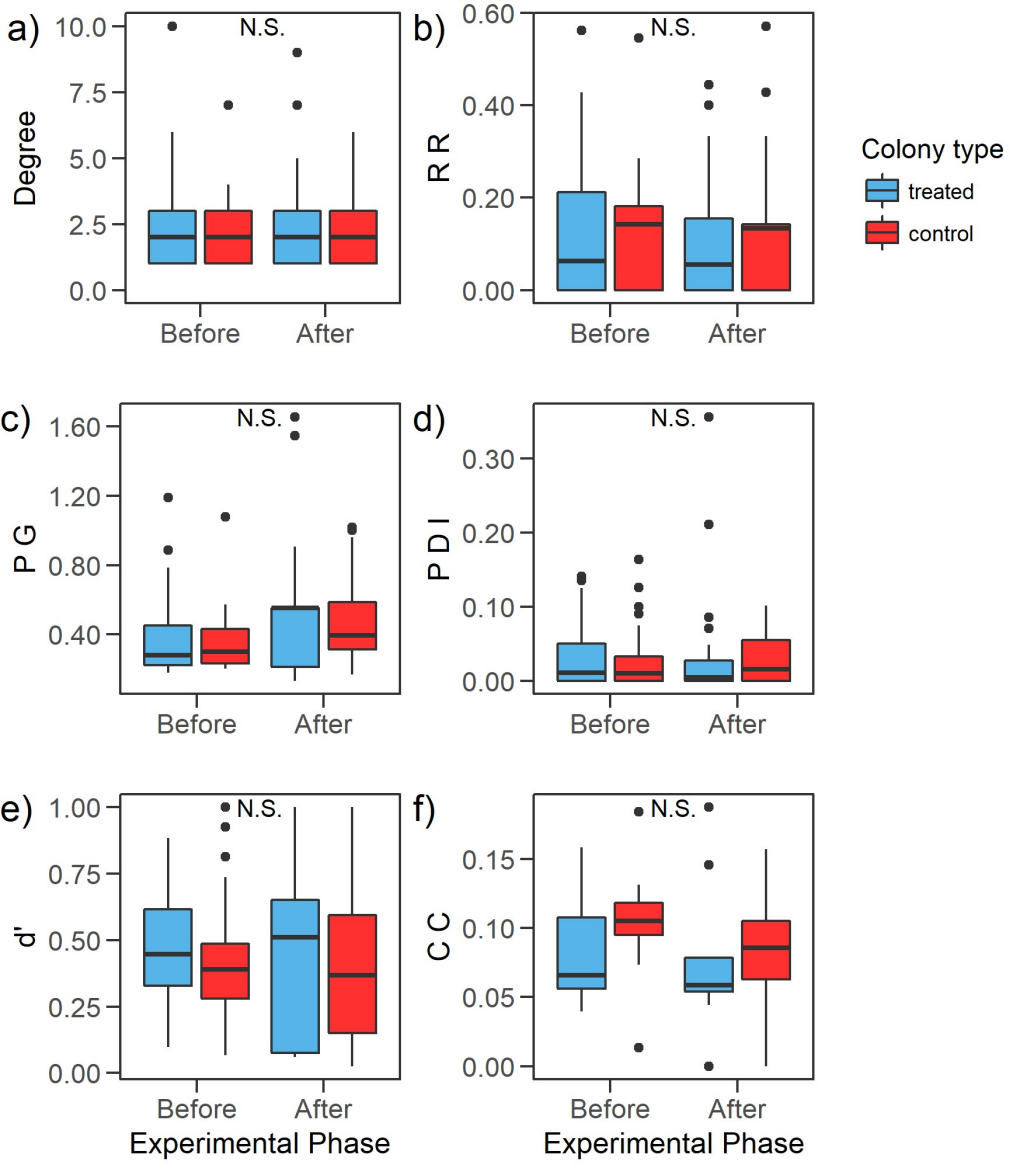
Node-level network indices describing aspects of foraging by individual bumblebees and their change during the experiment: (a) Degree, (b) RR: Resource Range, (c) PG: Proportional Generality, (d) PDI: Paired Difference Index, (e) d’: Complementary specialization, (f) CC: Closeness Centrality for plants (see methods). “N.S” signifies not statistically significant and the statistical outputs of the GLMMs are in Table 2.

The node level network analyses in the phase before removal revealed that Degree and PG were low but the plant’s Closeness Centrality was high and PDI and RR were both low, while d’ spanned over a wide range of the specialization-generalization gradient (Fig 1). Changes after treatment were generally not significant (Table 2 and Fig 1); only the PG index increased after workers removal (βafter− β_before_ = 0.241, p < 0.05) while it did not differ between treated and control colonies and the interaction between treated/control colonies and experimental phase were not significant predictors (Table 2 and Fig 1).

**Table 2:** Node—level indices tested for significant changes after halving the colony workforce by likelihood-ratio test of GLMM models with interaction between experimental phase (Before vs After) and T./C. (Treated vs Control colony). Statistical significance is highlighted in bold.

	Type	Experimental phase *x* T./C.	T./C.	Experimental phase
(a) Degree	Binary	**χ**^2^ = 0.442, df = 1, p = 0.506	**χ**^2^ = 0.043, df = 1, p = 0.835	**χ**^2^ = 0.202, df = 1, p = 0.652
(b) RR, Resource range	Binary	**χ**^2^ = 0.192, df = 1, p = 0.662	**χ**^2^ = 0.044, df = 1, p = 0. 834	**χ**^2^ = 0.920, df = 1, p = 0. 337
(c) PG, Proportional generality	Quantitative	**χ**^2^ = 0.177, df = 1, p = 0.674	**χ**^2^ = 0.041, df = 1, p = 0.840	**χ**^2^ = 5.438, df = 1, p = **0.012**
(d) PDI, Paired Difference Index	Quantitative	**χ**^2^ = 0.466, df = 1, p = 0.495	**χ**^2^ = 0.218, df = 1, p = 0.640	**χ**^2^ = 0.048, df = 1, p = 0.826
(e) d’, Complementary specialization	Quantitative	**χ**^2^ = 0.001, df = 1, p = 0.993	**χ**^2^ = 0.389, df = 1, p = 0.533	**χ**^2^ = 0.408, df = 1, p = 0.523
(f) Plants’ Closeness Centrality	Binary	**χ**^2^ = 3.427, df = 1, p = 0.064	**χ**^2^ = 0.001, df = 1, p = 0.985	**χ**^2^ = 0.214, df = 1, p = 0.644

**Fig 2:**
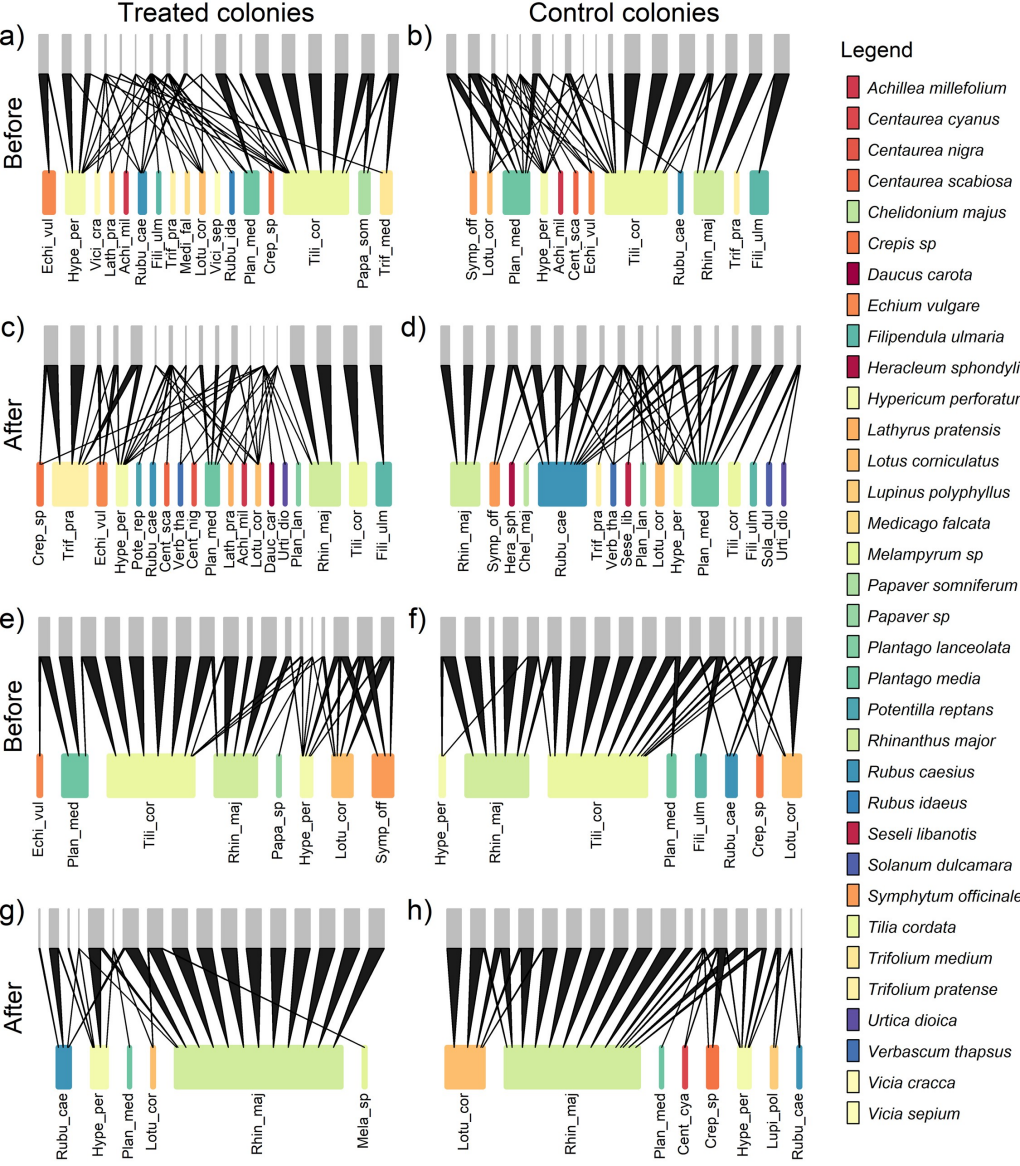
Bumblebee-plant networks during the experimental phases of before and after the workforce removal (Treated nest 1 in panels “a”, “c”; Control nest 3 in panels “b”, “d”, Treated nest 2 in panels “e”, “g”; Control nest 4 in panels “f”, “h”). For each network, the grey layer at the top represents individual bumblebees while the other layer indicate plants; for each plant species a specific colour is given and codes of 4 and 3 letters of their genus and species names respectively are used but full names are provided in the legend.

The binary indexes of the network-level analyses didn’t change significantly after treatment in the treated colonies. On the other hand, only two of the quantitative indexes (i.e., the Link Density and Vulnerability of plants) changed significantly over the study period (Table 3 and Fig 2).

**Table 3:** Network indices tested for change during the experimental phases (before and after the worker removal) by 10000 random permutational swaps of interactions between the networks before and after the treatment. Statistical significance is highlighted in bold. N_1_ and N_2_ indicate the treated nest’s identity.

	Type		Before	After	p	p in control
Link Density	Quantitative	N_1_ =	3	1.87	**0.006**	0.335
		N_2_ =	2.94	5.57	**0.043**	0.224
Connectance	Binary	N_1_ =	0.17	0.15	0.221	0.05
		N_2_ =	0.25	0.25	1	0.677
NODF	Binary	N_1_ =	17.21	11.55	0.054	0.482
		N_2_ =	7.68	7.96	0.926	0.625
Weighted NODF	Quantitative	N_1_ =	12.77	12.45	0.925	0.881
		N_2_ =	8.33	5.18	0.613	0.96
Modularity	Binary	N_1_ =	0.42	0.5	0.09	0.581
		N_2_ =	0.48	0.47	0.829	0.634
Weighted Modularity	Quantitative	N_1_ =	0.65	0.75	0.151	0.248
		N_2_ =	0.68	0.26	**0.015**	**0.046**
H2'	Quantitative	N_1_ =	0.85	0.92	0.365	0.362
		N_2_ =	0.87	0.83	0.768	0.31
Generality	Quantitative	N_1_ =	1.67	1.47	0.388	0.089
		N_2_ =	1.34	1.17	0.381	0.969
Vulnerability	Quantitative	N_1_ =	4.34	2.27	**0.004**	0.459
		N_2_ =	4.55	9.97	**0.032**	0.199

### Rate of workers leaving nest

**Fig 3:**
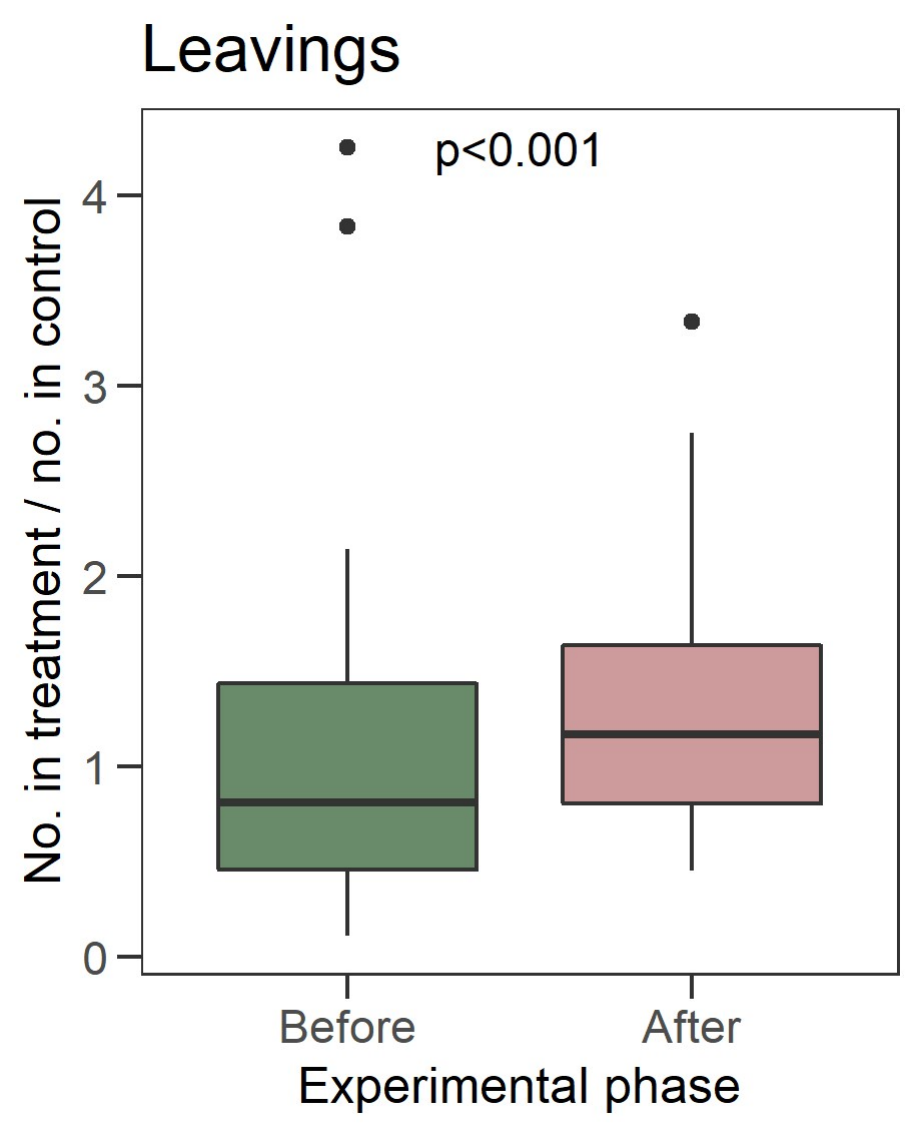
Number of workers leaving their nests per time unit (20 minutes long) proportionally to the control’s leaving during the same time units. Significance is tested with a GLMM (see methods).

After removing the workforce, the proportion of workers leaving the treated nests relative to the control nests’ foraging rate increased (Fig 3, the trend without the proportion to control’s foraging is in S2 Fig). Specifically, the treatment was a significant predictor of the number of workers leaving in the GLMM with an offset of the control’s leavings (βafter− β_before_ = 0.40, likelihood ratio test χ^2^ = 14.945, df = 1, p < 0.001).

### Pollen quantity

**Fig 4:**
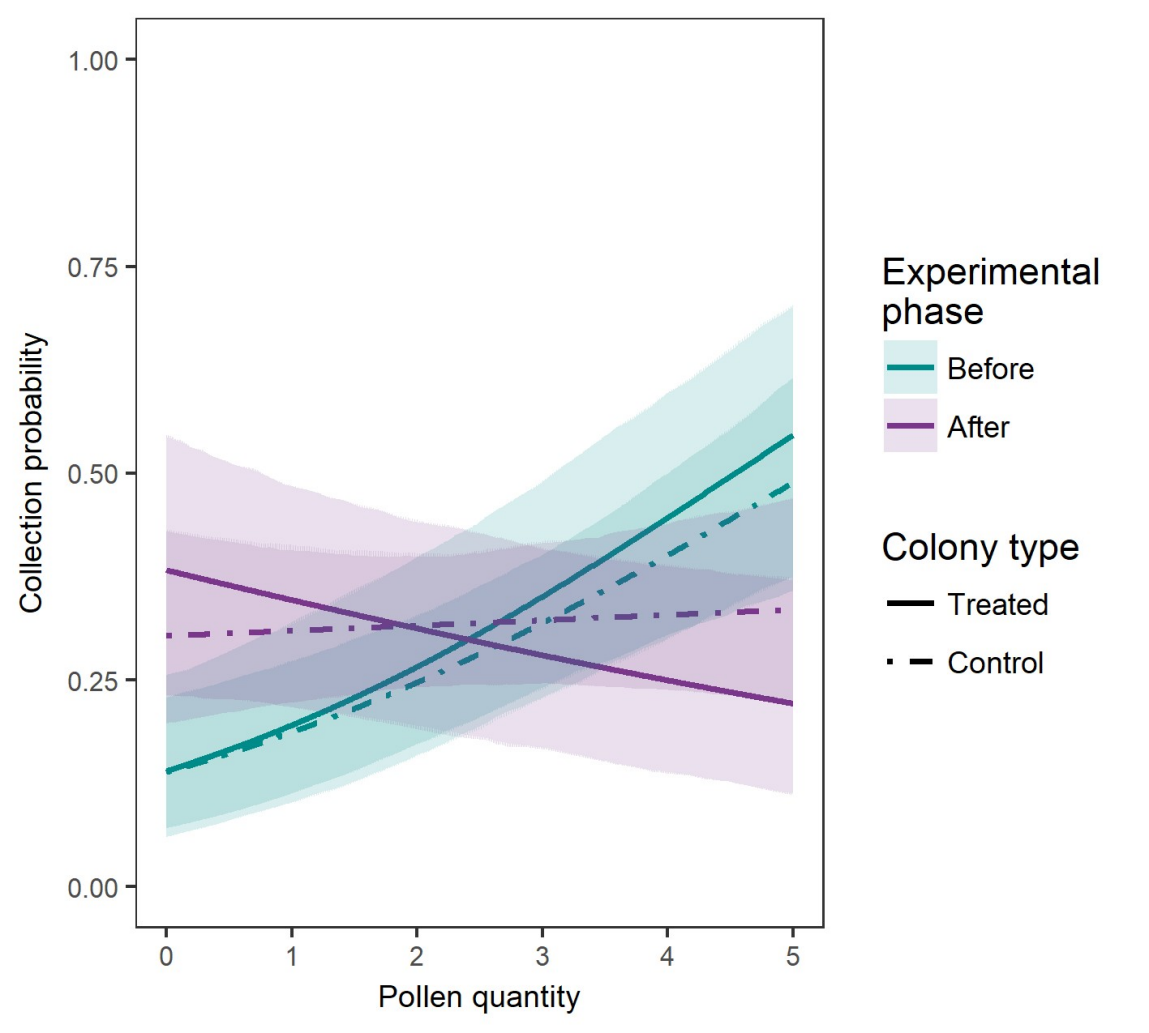
Probability of collecting pollen in relation to the traits of pollen quantity of the foraged plants during both experimental phases (“before” and “after” workforce reduction) in the treated and control colonies. The plot shows the estimated probabilities (lines) and the 95% confidence intervals (polygons).

Collection probability of individual plant species significantly depended on the interaction of the experimental phase with pollen quantity, as shown in Table 4 and Fig 4. While collection probability was positively related to pollen quantity in the period before workforce reduction (β_before_ = 0.399, p < 0.001), the relationship was negative in the period after workforce reduction (βafter− β_before_ = -0.554, p <0.001). However, this happened in both treated and control colonies, i.e. there was no significant effect of the treatment on the relationship between pollen quantity and collection probability (Table 4).

**Table 4:** Results of the likelihood ratio test of the logistic regression including the interactions between the experimental phase (Before vs After), the T./C. (Treated vs Control colony) and the pollen quality.

Variable	χ^2^	df	p
Experimental phase	0.001	1	0.976
Pollen quantity	10.731	1	**<0.01**
T./C.	0.0251	1	0.874
Experimental phase *x* Pollen quantity	23.541	1	**< 0.001**
Experimental phase *x* T./C.	0.893	1	0.345
Pollen quality *x* T./C.	0.660	1	0.417
Experimental phase *x* T./C. *x* Pollen quality	1.517	1	0.218

## Discussion

Previous studies on the foraging activity of bumblebees mainly focused on altering a diet and investigating adjustments in foraging in laboratory conditions [23,40,41]. Instead, we investigated how reductions in colony size would affect the resource utilization and the foraging behaviour of these key pollinators when free to forage in the field after a strong reduction of workforce. This is a novel aspect because, to our knowledge, only a few studies have previously investigated the effect of experimentally removing the bumblebees’ workforce exclusively on colony fitness [14], on the feeding of larvae [13] and on intra-colony task allocation [20].

In our study, we have used DNA metabarcoding to identify pollen, and this approach was reliable, because the plant list found in the pellets of our study matches other central European surveys [77,78]. Overall, the list of 34 plants found in the pollen samples retrieved from the individual foragers over the short time of our study highlights how polylectic bees normally rely on a wide set of flowering species [30,79]. Not only was the total plant diversity large, but our results also show that single foragers were indeed polylectic, as more than 60% of the pollen samples recovered from the bumblebees’ foragers were polyfloral, and this is consistent with the literature [78]. Stocking polyfloral pollen pellets in the nest is considered to be an adaptive advantage for overcoming the among-plants variability of pollen nutritional quality [30,80].

Our results support the hypothesis that bumblebee colonies may respond to the reduction of workforce by increasing the foraging activity of the remaining workers. We observed an increased rate of leaving the nest in the colonies that were subjected to the workforce removal, relative to control nests (Fig 3), which suggests an increase in the foraging effort of the colonies. Such an increased foraging could either result from the single foragers making more foraging bouts per unit time (allocating more energy in travelling), or, result from behavioural switching in the nest as an increase in the number of foraging workers relative to workers doing other behavioural tasks. A link between the foraging rate and the amount of pollen stored in the nest was found in honeybees, because they forage more frequently after reductions in the amount of stored pollen [44,81]. Similarly, the increased foraging observed in our experiment could result either in storing a higher amount of pollen in the nest or in storing an overall wider plant diversity, to compensate for the missing workers.

Plant choices for pollen collection were influenced by the plant’s pollen quantity as the bumblebees preferred pollen from plants that were high-ranked in the pollen-production database we have used (Fig 4) [74]. Nevertheless, in the experimental phase after workforce removal, plants producing a lower amount of pollen prevailed in the samples but without any emerging difference between control and treated colonies. The lack of difference between control and treated colonies indicates that this pattern is due to factors external to the colony, at least in the short term of the timing of our experimental manipulation. Thus, slight phenological changes of the plant assemblage at the study site determined the plant choices by the foragers. That the vegetation phenological changes played a role is also supported by the fact that the workers from all nests, both treated and control ones, collected a diversity of plants that was different between the “before-removal” phase and the “after-removal” phase (Table 1 and S1 Fig). Thus, it is possible that several plants shifted the status of the anthers’ maturation while still blooming during the time of the experiment, as it is common in plants [82].

The other hypothesis, i.e. that bumblebees would change the foraging strategies, was not observed in our data. Specifically, the node-level network indices revealed small and non-significant changes in the level of specialism/generalism, in the number of gathered plants, in the proportion of the available resources actually collected, and in the centrality in the plants in the network (Table 2 and Fig 1). Similarly, the diet breadth of bumblebees did also not expand and the bumblebee-plant network was not impacted by the workforce manipulation according to the indices of binary networks (based on presence/absence of interactions), and there were only minor changes in some quantitative network indices related to the quantity of the resources used by foragers (Table 3 and Fig 2). This result is particularly surprising, because it contradicts the expectations based on density-dependent foraging that should have taken place due to an altered intra-specific competition after the manipulation. That is, a higher abundance of foraging bumblebees (as when workforce is high) can force foragers to use more plants as a consequence of a faster depletion of the favourite resources and this implies a higher generalisation in resource use [23]. Conversely, our study did not find higher specialisation when foragers were few (i.e. during the “after” removal period in the treated nests). Our findings are supported by other choice experiments that showed a constancy in plant usage at higher bumblebee densities [43] as well as that higher forager density did not change bumblebees’ foraging behavioural traits [40]. For the fact that bumblebees are able to change behaviour [20], after removing workforce a given colony might have increased the fraction of foragers relative to other behavioural tasks. Although we do not have specific data for supporting or not supporting this, a behavioural switch could have prevented the expected variation of foragers density to take place after removal. The direct effect of such a behavioural switching and more generally whether or not pollinators forage according to density dependent mechanisms deserves further study in order to clarify how resources are collected in relation to forager density, at least under field conditions.

This constancy in the foraging networks after workforce reduction can be explained by some aspects of bumblebees’ biology. One possibility is that bumblebee foragers were subjected to the effects of majoring and of traplines, that means that the foragers would keep favouring a specific plant species or a plant individual based of a superior quality of the resources; however, this is contradicted by our results indicating that the plant diversity for each forager significantly changed between experimental phases and in fact the foraging routes can be readjusted quickly following changes in rewards [83,84]. Instead, a more plausible factor causing the observed constancy is that in contrast to honeybees, the bumblebees are primitively eusocial which implies that colonies’ performance tends to rely more on individual choices of single foragers than on social information [41,85] (the latter being the case of honeybees). This results from workers of *Bombus terrestris* having almost no contact with the larvae during their development [86], which could explain why some of our expectations were not confirmed. The lack of direct feedback between larvae and forager could even uncouple the foraging choices and the colony’s growth rate in the long term, as it was clearly shown that removing workforce results in having less progeny and of smaller size [14].

In this study, we used a relatively small number of colonies because field experiment with bumblebee colonies are logistically challenging, but we applied a strong reduction of workforce (50% workers removed), so we believe our study had enough power to detect the effects of workforce reduction on bumblebee foraging. In addition, accumulation curves revealed that an acceptable level of plant diversity was yielded from the pollen samples, so the composition of pollen collected by the bumblebees was well characterised. Nevertheless, we acknowledge that an experimental design that included more colonies would potentially strengthen the results we have found, as social insects show some variation in the life-history traits, in morphology, in the stage of the colony (e.g. the foundation stage vs. the ergonomic growth stage vs. the reproductive stage) and in the proportion of workers allocated to each behavioural syndrome in the nest [87]. This variation is essentially sourced from a combination of environment and genetics [88], and in commercial bumblebee colonies, experts indicate, the variation can be very high (e.g., different starting masses, number of brood, condition of brood, etc). Therefore, future experiments may include aspects not considered in our experiment, such as (a) to test several bumblebee species if possible, (b) apply the experimental workforce reductions in more colonies in different habitats and (c) consider colony-level covariates (colony mass before and during the experiment, number of workers, brood condition) because they can modulate colony’s responses; these can provide further support to what we have observed in our study and reveal additional novel aspects of responses of bumblebee colonies to workforce loss.

## Conclusions

By using DNA metabarcoding of pollen samples to overcome limitations of the morphological identification, this study investigated the effect of workforce decreases on the bumblebee foraging dynamics, on the chosen plant’s pollen-production traits and on the foraging rate, using an experimental manipulation in the field. Such a sudden decrease in colony size may occur under natural conditions due to multiple stressors (e.g., pesticide exposure, parasites, and diseases [39]). After applying a reduction of pollinator’s workforce, the bumblebees’ foraging strategies and the heterogeneity of collected resources were mostly constant, except for the increase in the colony’s foraging rate. Our results did not support the expected adaptability of foraging in terms of collected diversity, similarly to other studies (see [89] and references there). While the increase in foraging activity of the remaining workers maintained the intake of pollen by the colony in the short term, it is uncertain whether this would translate into a successful long-term recovery from the loss of workers.

## Supporting information

S1 Appendix DNA analyses and taxonomical assignments. This file includes the detailed methods for the DNA analyses, the bioinformatic processing and the taxonomical assignments.
(PDF)

S1 Table Checklist of flowering species at the study area with GenBank accession numbers and the source of the ITS2 sequences used for the taxonomical identification of OTUs. (PDF)

S2 Table List of plants found in the pollen pellets of each nest (NC: control nests, NT: treated nests) and experimental phase (B: before treatment, A: after treatment), those plants not included in the preliminary botanical survey are marked with *.
(PDF)

S1 Dataset Raw data of interactions between bumblebees and plants of each nest (NC: control nests, NT: treated nests), nest type (TREATED, UNTREATED) and treatment (B: before treatment, A: after treatment). Bumblebees in rows are coded as “BB” followed by an identification number. Plant names in columns are shortened.
(CSV)

S1 Fig Relative change in the amount of sequencing reads for each plant species during the experiment. (PDF)

S2 Fig Number of leavings for each 20 minute time units in (a) treatment and (b) control colonies during the experimental phases of before and after workforce removal.
(PDF)
