# Correction: Foraging strategies are maintained despite workforce reduction: A multidisciplinary survey on the pollen collected by a social pollinator

**DOI:** 10.1371/journal.pone.0227453

**Published:** 2019-12-30

**Authors:** Paolo Biella, Nicola Tommasi, Asma Akter, Lorenzo Guzzetti, Jan Klecka, Anna Sandionigi, Massimo Labra, Andrea Galimberti

The affiliation for the fifth author is incorrect. Jan Klecka is not affiliated with #1 but with #3: Czech Academy of Sciences, Biology Centre, Institute of Entomology, České Budějovice, Czech Republic.

## References

[1] Biella P, Tommasi N, Akter A, Guzzetti L, Klecka J, Sandionigi A, et al (2019) Foraging strategies are maintained despite workforce reduction: A multidisciplinary survey on the pollen collected by a social pollinator. PLoS ONE 14(11): e0224037 10.1371/journal.pone.0224037 31693676 PMC6834249

