# Supplementary material for: Foraging strategies are maintained despite workforce reduction: A multidisciplinary survey on the pollen collected by a social pollinator
Source: PLoS One. 2019 Nov 6;14(11):e0224037. doi: 10.1371/journal.pone.0224037 (PMC6834249; doi:10.1371/journal.pone.0224037)
Supplement: S1 Appendix — This file includes the detailed methods for the DNA analyses, the bioinformatic processing and the taxonomical assignments. (PDF) [file pone.0224037.s001.pdf]

Supporting Information

Biella P., Tommasi N., Akter A., Guzzetti L., Klecka J., Sandionigi A., Labra M., Galimberti A.. Foraging strategies are maintained despite workforce reduction: a multidisciplinary survey on the pollen collected by a social pollinator. PloS one

S1 Appendix

DNA analyses and taxonomical assignments

After DNA extraction, sequencing libraries for each sample were prepared following Illumina guidelines (16S Metagenomic Sequencing Library Preparation, Part #15044223 Rev. B) with modifications for ITS2 sequencing. The ITS2 primers S2F and S3R were used with the addition of the Illumina overhang adapter sequences. namely

S2F_Seq:

5’TCGTCGGCAGCGTCAGATGTGTATAAGAGACAGATGCGATACTTGGTGTGAAT 3'

S3R_Seq:

5’GTCTCGTGGGCTCGGAGATGTGTATAAGAGACAGGACGCTTCTCCAGACTACAAT 3'.

Before amplification, DNA extracts were normalized by means of quantitative real-time PCR (qPCR) Ct values with the same amplification primer pairs and the same protocols described in [1]. PCR reactions contained 12.5 µl of KAPA HiFi HotStart ReadyMix PCR Kit, 5 µl of each primer 1 µM (forward and reverse) and 2.5 µl DNA (maximum volume of DNA per sample with 5ng/µl DNA concentration). Samples were initially denatured at 94° C for 5 min, then amplified using 40 cycles at 94° C for 30 s, 56° C for 30 s, and 72° C for 45 s. A final extension (72°) of 10 min was performed at the end of the programme to ensure complete amplification. All PCR amplifications were prepared under an UV PCR cabinet to avoid contamination. The success of amplification was tested on a 1.5% agarose gel-electrophoresis. A 100 bp mass ladder (GeneDirex 100 bp DNA Ladder RTU, FroggaBio Inc., Toronto, ON, Canada) was used to confirm the successful normalization of the amplicon concentration within the samples.

Index PCR and library sequencing were performed through the Illumina MiSeq instrument using MiSeq Reagent Kit v3 (2 x 300-bp paired-end sequencing). The library preparation and the sequencing process were conducted at BMR Genomics (Padova, Italy). Raw Illumina reads were paired and pre-processed using *USEARCH* 8.0.1623 [2]. Reads were filtered out if ambiguous bases were detected and lengths were outside the bounds of 250 bp. Moreover, an expected error of 1 was used as an indicator of read accuracy. OTUs (Operational Taxonomic Units) were obtained using *--cluster_fast* algorithm from *VSEARCH.2* software (https://github.com/torognes/vsearch)[3] with a 99% sequence identity. The cluster centroid was chosen as the representative sequence of the cluster. The taxonomic assignment of the representative sequences was carried out using the *BLAST* algorithm [4] against the reference DNA barcoding dataset of the study area (see above), accepting only assignments with Max Identity and Query Coverage > 98%. OTUs representative sequences showing assignment values of Maximum Identity and Query Coverage < 98% with the use of this database were assigned using the GenBank NCBI database with the above-mentioned thresholds. Taxonomic assignment at a genus level were preferred instead of a species level if the queried OTUs resulted in a Max Identity and Query Coverage > 98% with several species of a given genus and co-occurring at the study site (in the case of the DNA barcoding reference dataset) or within the investigated geographic region, i.e South Czech Republic (in the case of NCBI queries).

Literature

1. Bruno A, Sandionigi A, Rizzi E, Bernasconi M, Vicario S, Galimberti A, et al. Exploring the under-investigated "microbial dark matter" of drinking water treatment plants. Scientific reports. 2017;7: 44350.

2. Edgar RC. Search and clustering orders of magnitude faster than BLAST. Bioinformatics. 2010;26: 2460–2461.

3. Rognes T, Flouri T, Nichols B, Quince C, Mahé F. VSEARCH: a versatile open source tool for metagenomics. PeerJ. 2016;4: e2584.

4. Altschul SF, Gish W, Miller W, Myers EW, Lipman DJ. Basic local alignment search tool. Journal of molecular biology. 1990;215: 403–410.
