# Supplementary material for: Foraging strategies are maintained despite workforce reduction: A multidisciplinary survey on the pollen collected by a social pollinator
Source: PLoS One. 2019 Nov 6;14(11):e0224037. doi: 10.1371/journal.pone.0224037 (PMC6834249; doi:10.1371/journal.pone.0224037)
Supplement: S1 Table — (PDF) [file pone.0224037.s002.pdf]

Supporting Information

Biella P., Tommasi N., Akter A., Guzzetti L., Klecka J., Sandionigi A., Labra M., Galimberti A.. Foraging strategies are maintained despite workforce reduction: a multidisciplinary survey on the pollen collected by a social pollinator. PloS one

Supporting Tables

S1 Table – Checklist of flowering species at the study area, including the sources of the reference ITS2 sequences (i.e., this study and GenBank NCBI) and the GenBank accession numbers of the sequenced plants.

FLORAL CHECKLIST					
ORDER	FAMILY	GENUS	SPECIES	ITS2 REFERENCE SEQUENCES	ACCESSION
Asterales	Asteraceae	*Achillea*	*Achillea millefolium* L.	This study	LS973890
Apiales	Apiaceae	*Aegopodium*	*Aegopodium podagraria* L.	Genbank	
Rosales	Rosaceae	*Agrimonia*	*Agrimonia eupatoria* L.	Genbank	
Lamiales	Lamiaceae	*Ajuga*	*Ajuga genevensis* L.	This study	LS973891
Asparagales	Amaryllidaceae	*Allium*	*Allium scorodoprasum* L.	Genbank	
Asparagales	Asparagaceae	*Anthericum*	*Anthericum ramosum* L.	Genbank	
Apiales	Apiaceae	*Anthriscus*	*Anthriscus sylvestris* L.	This study	LS973892
Fabales	Fabaceae	*Astragalus*	*Astragalus glycyphyllos* L.	This study	LS973893
Lamiales	Lamiaceae	*Ballota*	*Ballota nigra* L.	This study	LS973894
Asterales	Campanulaceae	*Campanula*	*Campanula patula* L.	This study	LS973895
Asterales	Campanulaceae	*Campanula*	*Campanula persicifolia* L.	This study	LS973896
Asterales	Campanulaceae	*Campanula*	*Campanula rapunculoides* L.	Genbank	
Asterales	Campanulaceae	*Campanula*	*Campanula vulgaris* L.	Genbank	
Asterales	Asteraceae	*Carduus*	*Carduus nutans* L.	This study	LS973897
Asterales	Asteraceae	*Centaurea*	*Centaurea jacea* L.	Genbank	
Asterales	Asteraceae	*Centaurea*	*Centaurea scabiosa* L.	This study	LS973898
Gentianales	Gentianaceae	*Centaurium*	*Centaurium erythraea* Rafn.	Genbank	
Asterales	Asteraceae	*Cichorium*	*Cichorium intybus* L.	Genbank	
Asterales	Asteraceae	*Cirsium*	*Cirsium arvense* L.	Genbank	
Asterales	Asteraceae	*Cirsium*	*Cirsium vulgare* (Savi) Ten.	Genbank	
Lamiales	Lamiaceae	*Clinopodium*	*Clinopodium vulgare* L.	Genbank	

Solanales	Convulvaceae	*Convolvolus*	*Convolvulus arvensis* L.	Genbank	
Apiales	Apiaceae	*Daucus*	*Daucus carota* L.	Genbank	
Caryophyllales	Caryophyllaceae	*Dianthus*	*Dianthus deltoides* L.	This study	LS973899
Asterales	Asteraceae	*Echinops*	*Echinops sp.* L.	This study	LS973900
Lamiales	Boraginaceae	*Echium*	*Echium vulgare* L.	This study	LS973901
Asterales	Asteraceae	*Erigeron*	*Erigeron annuus* L.	Genbank	
Brassicales	Brassicaceae	*Erysimum*	*Erysimum marschallianum* Andrz. ex M.Bieb.	This study	LS973902
Rubiales	Rubiaceae	*Galium*	*Galium album* Miller	Genbank	
Rubiales	Rubiaceae	*Galium*	*Galium aparine* L.	This study	LS973903
Rubiales	Rubiaceae	*Galium*	*Galium mollugo* L.	Genbank	
Rubiales	Rubiaceae	*Galium*	*Galium pumilum* Murray	Genbank	
Rubiales	Rubiaceae	*Galium*	*Galium verum* L.	Genbank	
Fabales	Fabaceae	*Genista*	*Genista tinctoria* L.	Genbank	
Gentianales	Gentianaceae	*Gentiana*	*Gentiana cruciata* L.	Genbank	
Geraniales	Geraniaceae	*Geranium*	*Geranium pyrenaicum* Burm.	This study	LS973904
Geraniales	Geraniaceae	*Geranium*	*Geranium sp.* L.	This study	LS973905
Geraniales	Geraniaceae	*Geranium*	*Geranium sylvaticum* L.	This study	LS973906
Rosales	Rosaceae	*Geum*	*Geum urbanum* L.	This study	LS973907
Malvales	Cistaceae	*Helianthemum*	*Helianthemum nummularium* L.	Genbank	
Apiales	Apiaceae	*Heracleum*	*Heracleum sphondylium* L.	This study	LS973908
Malpighiales	Hypericaceae	*Hypericum*	*Hypericum perforatum* L.	Genbank	
Asterales	Asteraceae	*Inula*	*Inula salicina* L.	This study	LS973909
Asterales	Asteraceae	*Senecio*	*Jacobaea vulgaris* Gaertn.	This study	LS973910
Dipsacales	Caprifoliaceae	*Knautia*	*Knautia arvensis* L.	This study	LS973911
Fabales	Fabaceae	*Lathyrus*	*Lathyrus heterophyllus* L.	This study	LS973912
Fabales	Fabaceae	*Lathyrus*	*Lathyrus pratensis* L.	This study	LS973913
Asterales	Asteraceae	*Leontodon*	*Leontodon hispidus* L.	This study	LS973914
Asterales	Asteraceae	*Leucanthemum*	*Leucanthemum vulgare* Lam.	Genbank	
Lamiales	Oleaceae	*Ligustrum*	*Ligustrum vulgare* L.	Genbank	
Liliales	Liliaceae	*Lilium*	*Lilium martagon* L.	Genbank	
Fabales	Fabaceae	*Lupinus*	*Lotus corniculatus* L.	Genbank	
Fabales	Fabaceae	*Lupinus*	*Lupinus polyphyllus* Lindl.	This study	LS973915
Malvales	Malvaceae	*Malva*	*Malva moschata* L.	Genbank	
Asterales	Asteraceae	*Matricaria*	*Matricaria chamomilla* L.	Genbank	
Fabales	Fabaceae	*Medicago*	*Medicago falcata* L.	This study	LS973916
Fabales	Fabaceae	*Medicago*	*Medicago lupulina* L.	Genbank	

Fabales	Fabaceae	*Medicago*	*Medicago sativa* L.	Genbank	
Lamiales	Orobanchaceae	*Melampyrum*	*Melampyrum nemorosum* L.	Genbank	
Lamiales	Orobanchaceae	*Melampyrum*	*Melampyrum pratense* L.	Genbank	
Fabales	Fabaceae	*Melilotus*	*Melilotus albus* Medik.	This study	LS973917
Fabales	Fabaceae	*Melilotus*	*Melilotus officinalis* L.	This study	LS973918
Boraginales	Boraginaceae	*Myosotis*	*Myosotis arvensis* L.	Genbank	
Fabales	Fabaceae	*Onobrychis*	*Onobrychis viciifolia* Scop.	This study	LS973919
Lamiales	Lamiaceae	*Origanum*	*Origanum vulgare* L.	Genbank	
Lamiales	Orobanchaceae	*Orobanche*	*Orobanche alba* Stephan ex Willd	Genbank	
Apiales	Apiaceae	*Pastinaca*	*Pastinaca sativa* L.	This study	LS973920
Asterales	Asteraceae	*Pilosella*	*Pilosella officinarum* Vaill.	This study	LS973921
Apiales	Apiaceae	*Pimpinella*	*Pimpinella saxifraga* L.	This study	LS973922
Lamiales	Plantaginaceae	*Plantago*	*Plantago lanceolata* L.	This study	LS973923
Lamiales	Plantaginaceae	*Plantago*	*Plantago media* L.	This study	LS973924
Fabales	Polygalaceae	*Polygala*	*Polygala vulgaris* L.	Genbank	
Rosales	Rosaceae	*Potentilla*	*Potentilla argentea* L.	Genbank	
Rosales	Rosaceae	*Potentilla*	*Potentilla erecta* L.	Genbank	
Rosales	Rosaceae	*Potentilla*	*Potentilla reptans* L.	Genbank	
Lamiales	Lamiaceae	*Prunella*	*Prunella vulgaris* L.	Genbank	
Ranunculales	Ranunculaceae	*Ranunculus*	*Ranunculus bulbosus* L.	Genbank	
Lamiales	Orobanchaceae	*Rhinanthus*	*Rhinanthus major* L.	This study	LS973925
Rosales	Rosaceae	*Rosa*	*Rosa canina* L.	This study	LS973926
Rosales	Rosaceae	*Rubus*	*Rubus caesius* L.	This study	LS973927
Rosales	Rosaceae	*Rubus*	*Rubus idaeus* L.	Genbank	
Rosales	Rosaceae	*Rubus*	*Rubus sp.* L.	This study	LS973928
Lamiales	Lamiaceae	*Salvia*	*Salvia verticillata* L.	This study	LS973929
Dipsacales	Caprifoliaceae	*Sambucus*	*Sambucus nigra* L.	This study	LS973930
Rosales	Rosaceae	*Sanguisorba*	*Sanguisorba minor* Scop.	Genbank	
Asterales	Asteraceae	*Scorzoneroides*	*Scorzoneroides autumnalis* L.	Genbank	
Fabales	Fabaceae	*Securigera*	*Securigera varia* L.	Genbank	
Saxifragales	Crassulaceae	*Sedum*	*Sedum sexangulare* L.	Genbank	
Apiales	Apiaceae	*Seseli*	*Seseli libanotis* L.	This study	LS973931
Lamiales	Lamiaceae	*Stachys*	*Stachys palustris* L.	This study	LS973932
Caryophyllales	Caryophyllaceae	*Stellaria*	*Stellaria graminea* L.	This study	LS973933
Dipsacales	Caprifoliaceae	*Symphoricarpos*	*Symphoricarpos albus* L.	Genbank	
Lamiales	Boraginaceae	*Symphytum*	*Symphytum officinale* L.	This study	LS973934

Asterales	Asteraceae	*Tancetum*	*Tanacetum vulgare L.*	This study	LS973935
Lamiales	Lamiaceae	*Teucrium*	*Teucrium chamaedrys* L.	Genbank	
Lamiales	Lamiaceae	*Thymus*	*Thymus pulegioides* L.	Genbank	
Malvales	Malvaceae	*Tilia*	*Tilia cordata* Mill.	This study	LS973936
Asterales	Asteraceae	*Tragopogon*	*Tragopogon pratensis* L.	This study	LS973937
Fabales	Fabaceae	*Trifolium*	*Trifolium arvense* L.	This study	LS973938
Fabales	Fabaceae	*Trifolium*	*Trifolium aureum* Pollich	This study	LS973939
Fabales	Fabaceae	*Trifolium*	*Trifolium medium* L.	This study	LS973940
Fabales	Fabaceae	*Trifolium*	*Trifolium montanum* L.	Genbank	
Fabales	Fabaceae	*Trifolium*	*Trifolium pratense* L.	This study	LS973941
Fabales	Fabaceae	*Trifolium*	*Trifolium repens* L.	Genbank	
Asterales	Asteraceae	*Tripleurospermum*	*Tripleurospermum inodorum* L.	Genbank	
Scrophulariales	Scrophulariaceae	*Verbascum*	*Verbascum chaixii* Vill.	This study	LS973942
Scrophulariales	Scrophulariaceae	*Verbascum*	*Verbascum lychnitis* L.	Genbank	
Scrophulariales	Scrophulariaceae	*Veronica*	*Veronica teucrium* L.	Genbank	
Fabales	Fabaceae	*Vicia*	*Vicia cracca* L.	Genbank	
Fabales	Fabaceae	*Vicia*	*Vicia sativa* L.	Genbank	
Fabales	Fabaceae	*Vicia*	*Vicia sepium* L.	This study	LS973943
Fabales	Fabaceae	*Vicia*	*Vicia tetrasperma* L.	Genbank	
