# Supplementary material for: Foraging strategies are maintained despite workforce reduction: A multidisciplinary survey on the pollen collected by a social pollinator
Source: PLoS One. 2019 Nov 6;14(11):e0224037. doi: 10.1371/journal.pone.0224037 (PMC6834249; doi:10.1371/journal.pone.0224037)
Supplement: S2 Table — List of plants found in the pollen pellets of each nest (NC: control nests, NT: treated nests) and experimental phase (B: before treatment, A: after treatment), those plants not included in the preliminary botanical survey are marked with *. (PDF) [file pone.0224037.s003.pdf]

Supporting Information

Biella P., Tommasi N., Akter A., Guzzetti L., Klecka J., Sandionigi A., Labra M., Galimberti A.. Foraging strategies are maintained despite workforce reduction: a multidisciplinary survey on the pollen collected by a social pollinator. PloS one

Supporting Tables

S2 Table - List of plants found in the pollen pellets of each nest (NC: control nests, NT: treated nests) and experimental phase (B: before treatment, A: after treatment), those plants not included in the preliminary botanical survey are marked with *.

ORDER	FAMILY	GENUS	SPECIES	ABBREVIATION	NC 1 B	NC1 A	NT 2 B	NT 2 A	NC4 B	NC4 A	NT3 B	NT3 A
Asterales	Asteraceae	*Achillea*	*Achillea millefolium* L.	Achi_mil	X		X	X				
Asterales	Asteraceae	*Centaurea*	*Centaurea cyanus* L.*	Cent_cya						X		
Asterales	Asteraceae	*Centaurea*	*Centaurea nigra* L.*	Cent_nig				X				
Asterales	Asteraceae	*Centaurea*	*Centaurea scabiosa* L.	Cent_sca	X			X				
Ranunculales	Papaveraceae	*Chelidonium*	*Chelidonium majus* L.*	Chel_maj		X						
Asterales	Asteraceae	*Crepis*	*Crepis sp.* L.*	Crep_sp			X	X	X	X		
Apiales	Apiaceae	*Daucus*	*Daucus carota* L.	Dauc_car				X				
Lamiales	Boraginaceae	*Echium*	*Echium vulgare* L.	Echi_vul	X		X	X			X	
Rosales	Rosaceae	*Filipendula*	*Filipendula ulmaria* L.*	Fili_ulm	X	X	X	X	X			
Apiales	Apiaceae	*Heracleum*	*Heracleum sphondylium* L.	Hera_sph		X			X			
Malpighiales	Hypericaceae	*Hypericum*	*Hypericum perforatum* L.	Hype_per	X	X	X	X		X	X	X
Fabales	Fabaceae	*Lathyrus*	*Lathyrus pratensis* L.	Lath_pra			X	X				
Fabales	Fabaceae	*Lotus*	*Lotus corniculatus* L.	Lotu_cor	X	X	X	X	X	X	X	X
Fabales	Fabaceae	*Lupinus*	*Lupinus polyphyllus* Lindl.	Lupi_pol						X		
Fabales	Fabaceae	*Medicago*	*Medicago falcata* L.	Medi_fal			X					
Lamiales	Orobanchaceae	*Melampyrum*	*Melampyrum sp.* L.	Mela_sp								X
Ranunculales	Papaveraceae	*Papaver*	*Papaver somniferum* L.*	Papa_som			X					
Ranunculales	Papaveraceae	*Papaver*	*Papaver sp.* L.*	Papa_sp							X	
Lamiales	Plantaginaceae	*Plantago*	*Plantago lanceolata* L.	Plan_lan		X		X				
Lamiales	Plantaginaceae	*Plantago*	*Plantago media* L.	Plan_med	X	X	X	X	X	X	X	X
Rosales	Rosaceae	*Potentilla*	*Potentilla reptans* L.	Pote_rep				X				
Lamiales	Orobanchaceae	*Rhinanthus*	*Rhinanthus major* L.	Rhin_maj	X	X		X	X	X	X	X
Rosales	Rosaceae	*Rubus*	*Rubus caesius* L.	Rubu_cae	X	X	X	X	X	X		X
Rosales	Rosaceae	*Rubus*	*Rubus idaeus* L.	Rubu_ida			X					
Apiales	Apiaceae	*Seseli*	*Seseli libanotis* L.	Sese_lib		X						

Solanales	Solanaceae	*Solanum*	*Solanum dulcamara* L.*	Sola_dul		*X*						
Lamiales	Boraginaceae	*Symphytum*	*Symphytum officinale* L.	Symp_off	*X*	*X*					*X*	
Malvales	Malvaceae	*Tilia*	*Tilia cordata* Mill.	Tili_cor	*X*	*X*	*X*	*X*	*X*		*X*	
Fabales	Fabaceae	*Trifolium*	*Trifolium medium* L.	Trif_med			*X*					
Fabales	Fabaceae	*Trifolium*	*Trifolium pratense* L.	Trif_pra	*X*	*X*	*X*	*X*				
Urticales	Urticaceae	*Urtica*	*Urtica dioica* L.*	Urti_dio		*X*		*X*				
Scrophulariales	Scrophulariaceae	*Verbascum*	*Verbascum thapsus* L.*	Verb_tha		*X*		*X*				
Fabales	Fabaceae	*Vicia*	*Vicia cracca* L.	Vici_cra			*X*					
Fabales	Fabaceae	*Vicia*	*Vicia sepium* L.	Vici_sep			*X*					
