# Supplementary material for: Foraging strategies are maintained despite workforce reduction: A multidisciplinary survey on the pollen collected by a social pollinator
Source: PLoS One. 2019 Nov 6;14(11):e0224037. doi: 10.1371/journal.pone.0224037 (PMC6834249; doi:10.1371/journal.pone.0224037)

Supporting Information

Biella P., Tommasi N., Akter A., Guzzetti L., Klecka J., Sandionigi A., Labra M., Galimberti A.. Foraging strategies are maintained despite workforce reduction: a multidisciplinary survey on the pollen collected by a social pollinator. PloS one

Supporting Figures

S1 Figure – Relative change in sequencing reads for each plant species during the experiment.

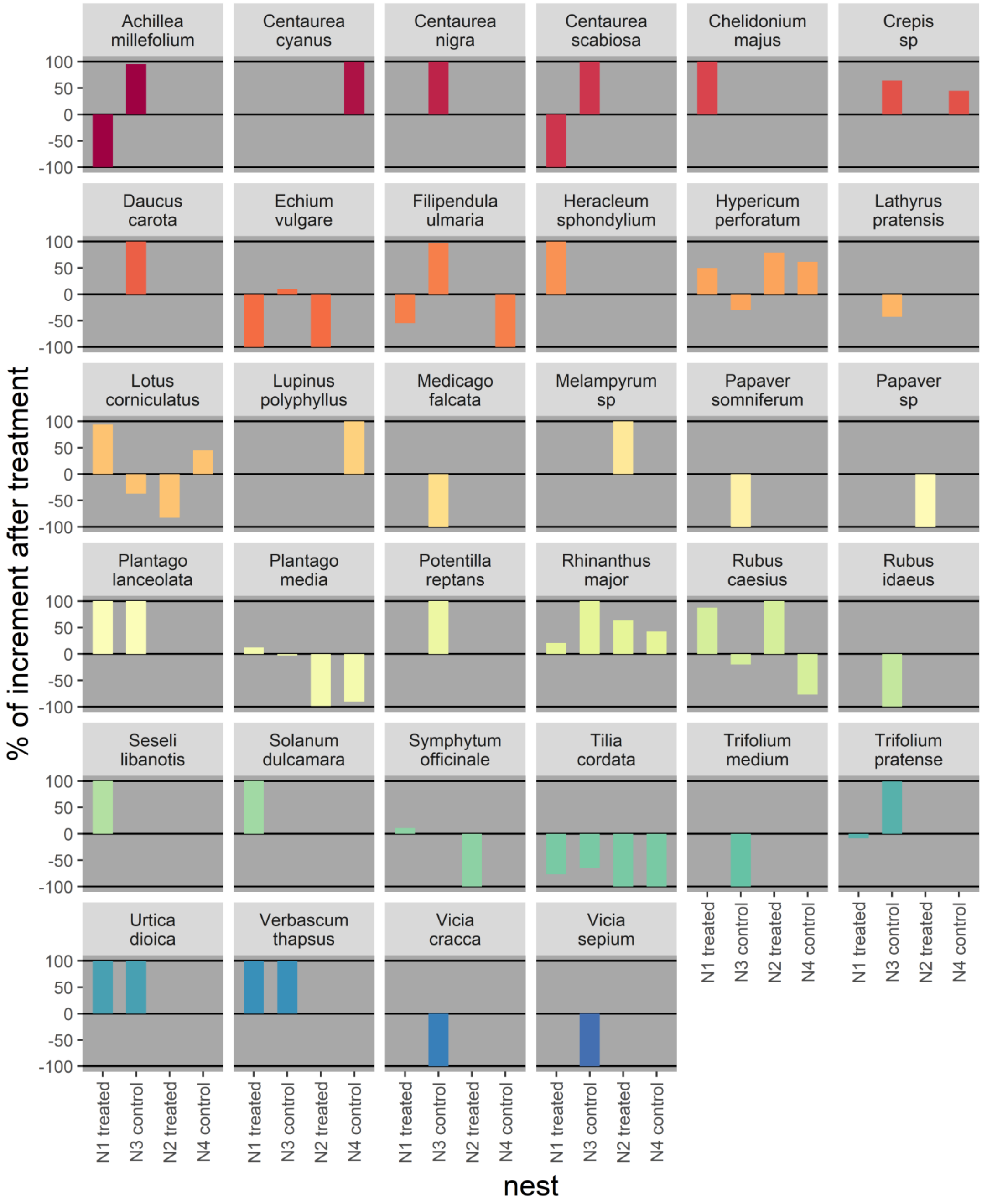

Supplement: S1 Fig — (PDF) [file pone.0224037.s005.pdf]
