# Supplementary material for: Foraging strategies are maintained despite workforce reduction: A multidisciplinary survey on the pollen collected by a social pollinator
Source: PLoS One. 2019 Nov 6;14(11):e0224037. doi: 10.1371/journal.pone.0224037 (PMC6834249; doi:10.1371/journal.pone.0224037)

Supporting Information

Biella P., Tommasi N., Akter A., Guzzetti L., Klecka J., Sandionigi A., Labra M., Galimberti A.. Foraging strategies are maintained despite workforce reduction: a multidisciplinary survey on the pollen collected by a social pollinator. PloS one

Supporting Figures

S2 Figure – Number of leavings and of returns for each 20 minutes times units in (a, b) treatment and (c, d) control colonies during the experimental phases of before and after workforce removal.

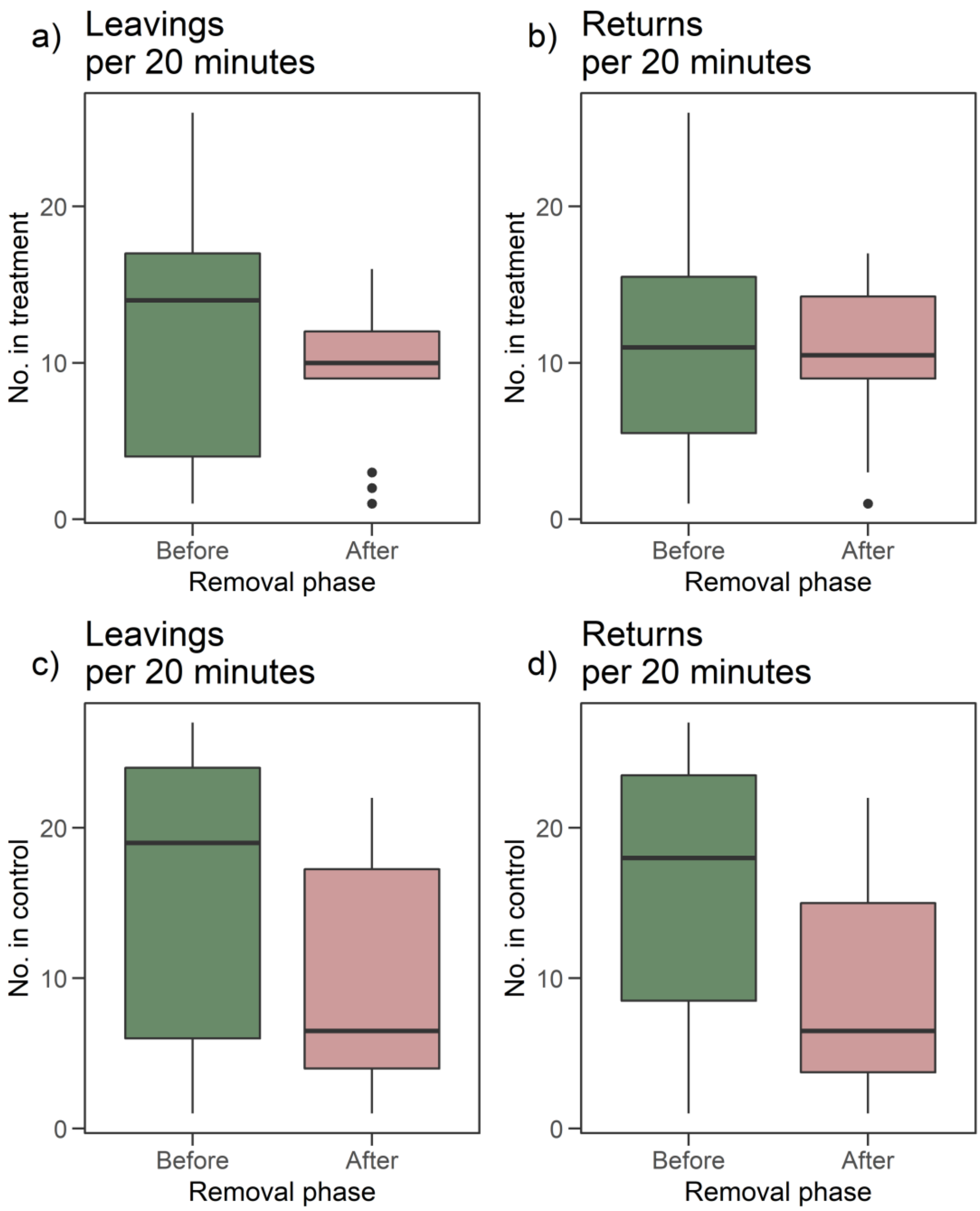

Supplement: S2 Fig — Number of leavings for each 20 minute time units in (a) treatment and (b) control colonies during the experimental phases of before and after workforce removal. (PDF) [file pone.0224037.s006.pdf]
